# How Elite Athletes with a Spinal Cord Injury Sweat during Exercise—An Exploratory Study

**DOI:** 10.3390/sports12030081

**Published:** 2024-03-14

**Authors:** Anneke Hertig-Godeschalk, Claudio Perret

**Affiliations:** 1 Institute of Sports Medicine, Swiss Paraplegic Centre, 6207 Nottwil, Switzerland; 2 Swiss Paraplegic Research, 6207 Nottwil, Switzerland; 3 Faculty of Health Sciences and Medicine, University of Lucerne, 6005 Lucerne, Switzerland

**Keywords:** para-athlete, paraplegia, tetraplegia, Paralympics, thermoregulation, exercise, hydration

## Abstract

Background: Sweat and thermal responses in individuals with spinal cord injury (SCI) are impaired depending on lesion characteristics. This is particularly problematic for athletes and may ultimately lead to reduced performance. This exploratory study investigated the feasibility of field-usable methods to objectively collect data relevant to sweat response in elite athletes with SCI. Differences in sweat response were also evaluated for different athlete characteristics. Methods: Measurements were performed during exercise and included core temperature (Tc), heart rate, urine specific gravity, fluid intake, sweat rate, and sweat electrolyte concentration. Differences for sex, lesion level (tetraplegia versus paraplegia), motor impairment (complete versus incomplete), and sport type (endurance versus team/skill) were evaluated. Results: Fifteen athletes (median (Q1–Q3) age, 30 (28–36) years; three females; 11 with complete lesions) were included. Endurance athletes were measured during indoor performance tests (n = 10), whereas team/skill athletes were measured during training sessions (n = 5). In the mixed exercise intensities, the average Tc was 37.7 (37.3–37.8) °C and the average heart rate was 126 (100–146) bpm. Dehydration, defined as a urine specific gravity > 1.020 ng/mL, was prevalent in six athletes before exercise and in five athletes after exercise. The sweat rate was lower in athletes with tetraplegia (*p* = 0.02) and in team/skill athletes (*p* = 0.008). Conclusions: Collecting sweat and thermal response data from athletes with SCI in the field is feasible. Given the suboptimal hydration status of many athletes, raising awareness of the importance of hydration seems valuable.

## 1. Introduction

Evidence from non-disabled athletes shows that personalized hydration recommendations based on sweat rate, fluid intake, exercise intensity, and environmental conditions are essential to optimize performance [1]. For athletes with spinal cord injury (SCI), the situation is even more complex as lesion characteristics must also be considered. Depending on the level and completeness of the SCI, vasomotor and sudomotor regulation are proportionally affected [2,3,4]. The higher the lesion level, the more the thermoregulatory system is impaired. Individuals with SCI may have a reduced ability to sweat and a higher core temperature (Tc) compared to non-disabled individuals [4,5]. This can lead to impaired performance and an increased risk of heat injury, particularly during exercise in warm conditions [4,5,6]. In addition to specific cooling techniques, fluid replacement can mitigate the negative effects of the impaired thermoregulatory response on performance [4,5,6]. Both overhydration—in individuals with low sweat rates—and dehydration—in individuals with higher sweat rates—are known to occur in athletes with SCI [7].

Personalized hydration recommendations require objective measurements that can also be performed in the field. Conducting studies in individuals with SCI can be challenging due to practical issues, the small population available, and individual heterogeneity [8]. Most studies evaluating sweat rate and electrolyte concentrations in athletes with SCI have been conducted either in the laboratory or in team sports such as basketball and rugby [4]. Studies in other sports, including handcycling, are rare. Especially in endurance sports such as wheelchair racing or handcycling, the duration of exertion and heat exposure is longer, making adequate fluid intake even more important. In this exploratory study, we investigated the feasibility of field-usable methods to collect sweat rate and electrolyte concentration data during exercise in elite athletes with SCI. We hypothesized that our methods should be feasible for data collection in most athletes with SCI. In addition, we evaluated differences in individual characteristics, including lesion level and sport type.

## 2. Materials and Methods

### 2.1. Study Design and Population

This observational study was designed to evaluate objective data collection methods that can be used in field settings. Therefore, sample size calculation was not performed. We used a cross-sectional study design and adhered to the STROBE guidelines (Supplementary Table S1) [9]. This study was conducted at a sports medicine center specializing in supporting elite wheelchair athletes, including training consultation and performance testing. Data were collected between June 2022 and February 2023. All study procedures and data collection were performed by trained personnel.

Athletes affiliated with the sports medicine center were invited to participate in this study. The following eligibility criteria were applied: currently active in (inter)national competitions, having a Paralympic classification, having a traumatic or non-traumatic SCI, and aged between 18 and 55 years (as this age range encompasses the Swiss elite squad). This study was conducted in accordance with the Declaration of Helsinki and Swiss law and was approved by the local ethics committee (EKNZ, Basel, Switzerland, project-ID: 2022-00886). Written informed consent was obtained from all athletes. No financial compensation was provided for participation in this study.

### 2.2. Data Collection

The following individual characteristics were collected: demographics (sex, age, body mass index (BMI)), lesion (cause, duration, level, American Spinal Injury Association Impairment Scale (AIS)), maximum heart rate (measured during a previous performance test at the sports medicine center), years active as an elite athlete, sport type, and weekly training (duration and number of sessions). Athletes were asked to rate their (subjective) sweat response above and below the lesion level as “normal”, “increased”, “decreased”, or “failing”.

All measurements were performed in the sport-specific wheelchair during either regular training sessions or performance tests. Athletes were asked to void their bladder before the start of the exercise. Ambient temperature and humidity were measured at the beginning of the exercise (HT30 Heat Stress WBGT Meter, Extech Instruments, Teledyne FLIR, Wilsonville, OR, USA). After cleaning the respective skin location with an alcohol wipe, absorbent patches (Tegaderm + Pad 3582 (pad size 2.5 × 4 cm), 3M, St. Paul, MN, USA) were placed on the forehead, left and right scapulae, dorsal mid-forearms, and shins following Baker et al. (2018) [10] (Supplementary Figure S1). After exercise, the patches were removed from the skin. Next, the absorbent pad was separated from the adhesive plaster and placed into a syringe using tweezers. The available sweat was squeezed out of the syringe and the electrolyte concentrations of sodium (Na^+^) and potassium (K^+^) were assessed using ion-selective meters (LAQUAtwin Na-11 and K-11, Horiba, Kyoto, Japan).

The following parameters were collected at the beginning (“pre-exercise”) and at the end (“post-exercise”) of the exercise. Urine specific gravity (USG) was measured with a refractometer (ORF-2UM, Kern & Sohn GmbH, Balingen, Germany). Body mass (BM) was measured using a wheelchair-accessible scale, which allowed the athlete’s weight to be calculated separately from the wheelchair. During BM measurements, the athlete wore minimal clothing (bare chest for males and sports bra for females). After exercise, all sweat was toweled off before BM was measured. For athletes who consumed fluids during exercise, the type of drink and the weight of the drink bottle pre- and post-exercise were recorded. Solid food intake and toilet breaks were also recorded. A heart rate sensor (TP5, Cardiosport, Hampshire, UK) was worn on the chest to continuously assess heart rate during exercise. An ingestible electronic capsule (Bodycap, Hérouville Saint-Clair, France) was used to continuously assess Tc during exercise. These Tc data were used for the main analyses. Corresponding with previous studies in individuals with SCI, the capsule was swallowed at a minimum of eight hours before the start of exercise [11,12]. A wearable sensor (CORE, greenTEG, Zürich, Switzerland) was attached to the heart rate chest strap and used to provide additional core and skin temperature measurements. The same sensor was also used to measure skin temperature on the shin. Post-exercise, Borg Rating of Perceived Exertion (RPE) was assessed on a 15-point scale ranging from 6 (“no exertion at all”) to 20 (“maximum effort”) [13].

### 2.3. Data Preparation

The neurological level of injury, defined as the highest intact sensorimotor level, was categorized as “tetraplegia” (C1–C8) or “paraplegia” (T1 or lower) [14]. The AIS was used to categorize the degree of motor impairment as a “complete” (A or B) or “incomplete” (C or D) lesion [14]. Athletes participating in either cycling or wheelchair racing were grouped as “endurance athletes”, while athletes participating in basketball, rugby, and alpine skiing were grouped as “team/skill athletes”. Average, maximum, and minimum values were determined for heart rate, Tc, and skin temperature. Relative heart rate was calculated as the average heart rate relative to the maximum heart rate. USG was categorized as “hydrated” (≤1.020 g/mL) or “dehydrated” (>1.020 g/mL) [15]. Absolute BM difference and fluid intake were assessed by the difference in BM and bottle weight pre- versus post-exercise. Relative BM difference was calculated as BM difference in relation to pre-exercise BM. Fluid intake (L/h) during the measurement was calculated as fluid intake relative to exercise duration. Indirect sweat rate (L/h) was calculated as BM difference plus fluid intake minus urine loss, relative to the exercise duration [16]. Electrolyte loss (mg/h) was estimated as electrolyte concentration multiplied by sweat rate.

### 2.4. Data Analysis

The Shapiro–Wilk test indicated that the primary outcome variable sweat rate was non-normally distributed (*p* = 0.43). Also, due to the small sample size, we decided to apply non-parametric methods. Data are described by reporting the median and interquartile range (Q1–Q3). Differences in electrolyte concentration between the left and right sides of the body for each skin location, and between the core temperature of the pill and sensor were tested using Wilcoxon signed-rank tests. Differences in fluid intake, sweat rate, USG, and electrolyte concentration between different athlete characteristics (sex, lesion cause, lesion level, lesion impairment, and sport type) were tested using the two-sample Wilcoxon rank-sum (Mann–Whitney). Effect sizes for non-parametric tests were calculated using r = z/√N, with an r-value between 0.1 and 0.3 indicating a small effect size, between 0.3 and 0.5 indicating a medium effect size, and above 0.5 indicating a large effect size [17]. The correlations between sweat rate versus electrolyte concentration and heart rate were assessed using Spearman’s correlation. A two-tailed *p*-value of 0.05 or less was considered statistically significant. No imputation of or deletion of cases with missing data was performed. Analyses were performed with Stata (StataCorp. 2017, Stata Statistical Software: Release 17.0. StataCorp LLC, College Station, TX, USA).

## 3. Results

Of the fifteen athletes included in this study, five had competed at an elite level for one year and ten had competed at an elite level for 3 to 30 years (Table 1). Measurements were performed in indoor training facilities under controlled conditions. Pre-exercise conditions were as follows: median (Q1–Q3) ambient temperature, 21 (21–22) °C; humidity, 34 (27–36)%. Measurements were performed over a range of exercise intensities, as indicated by the wide range of heart rates (Table 2). The endurance athletes were assessed during performance tests on a handcycle or racing wheelchair (n = 10). The team/skill athletes were assessed either during endurance arm-crank training (n = 3), basketball (n = 1), or rugby (n = 1) training with a typical intermittent characteristic. Measurements had a duration of 68 (42–78) min. Athletes rated the RPE of the exercise as 16 (15–17), with corresponding relative heart rates of 73 (69–76)% (Table 2A). Above the lesion level, athletes rated their sweat response as normal (n = 7), increased (n = 5), or absent (n = 3). Below the lesion level, athletes rated their sweat response as normal (n = 5), decreased (n = 4), absent (n = 4), or increased (n = 2).

Measurements could be successfully performed on most athletes, provided there was access to a wheelchair-accessible scale to allow accurate BM measurements. There were missing data for BM (n = 1), USG (pre-exercise n = 2, post-exercise n = 1), Tc (n = 2), and electrolyte concentrations (head n = 6, scapula n = 4, arm n = 5, shin n = 10). Reasons for missing data included scale or sensor malfunction, or insufficient urine or sweat production.

**Figure 1:**
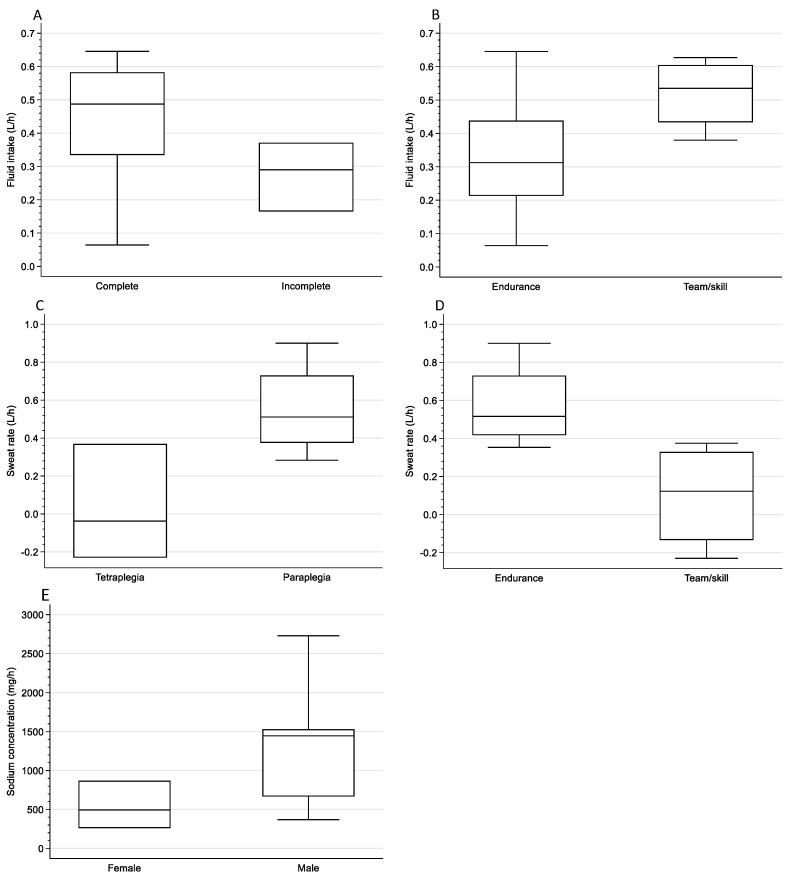
Boxplots for fluid intake by (**A**) impairment (*p* = 0.21, r = 0.40) and (**B**) sport type (*p* = 0.11, r = 0.40); sweat rate by (**C**) lesion level (*p* = 0.02, r = 0.60) and (**D**) sport type (*p* = 0.008, r = 0.68); (**E**) sodium concentration at the left scapula by sex (*p* = 0.12, r = 0.54).

When comparing athletes with tetraplegia versus paraplegia, average (37.9 (36.9–38.8) versus 37.3 (37.3–37.8) °C, *p* = 0.92, r = 0.10) and maximum (38.7 (37.6–39.8) versus 38.1 (37.9–38.6) °C, *p* = 0.74, r = 0.11) Tc did not differ (*p* ≥ 0.74). The average core temperature measured by the pill was comparable to that of the wearable sensor (37.7 (37.3–37.8) versus 37.8 (37.4–38.0) °C, *p* = 0.13, r = 0.43, Supplementary Table S2). Most athletes (n = 9) consumed water during exercise, while the remaining athletes consumed either a sports drink (n = 3) or nothing (n = 3). Fluid intake ranged from 0.06 to 0.65 L/h. None of the athletes consumed non-liquid foods during exercise. One athlete used the toilet during exercise. Dehydration was prevalent pre-exercise in six athletes, in more team/skill athletes or athletes with tetraplegia. Post-exercise, dehydration was prevalent in five athletes, in more endurance athletes or athletes with paraplegia. A trend with a large effect size was found for pre-exercise USG by sport type, with endurance athletes having better USG compared to team/skill athletes (*p* = 0.07, r = 0.51, Table 2A). The relative difference in BM ranged from −0.9 to 1.6%. A medium effect size was found for fluid intake by impairment, with athletes with a complete lesion having a higher fluid intake than athletes with an incomplete lesion (0.49 (0.42–0.57) versus 0.39 (0.28–0.73) L/h, *p* = 0.21, r = 0.40, Figure 1A). A trend with a medium effect size was found for fluid intake by sport type, with endurance athletes having a lower fluid intake than team/skill athletes (*p* = 0.11, r = 0.40, Table 2A, Figure 1B). Sweat rate was lower in athletes with tetraplegia compared to those with paraplegia (−0.04 (−0.23–0.37) versus 0.51 (0.38–0.73) L/h, *p* = 0.02, r = 0.60, Figure 1C). Sweat rate was lower in team/skill athletes compared to endurance athletes (*p* = 0.008, r = 0.68, Table 2A, Figure 1D). Athletes with a higher average heart rate had a higher sweat rate (r_s_ = 0.61, *p* = 0.021, Supplementary Figure S2A). Electrolyte concentrations could only be analyzed in athletes with paraplegia. There were no differences in electrolyte concentrations between the left and right sides of the body (Table 2B). Athletes with higher sodium concentrations had a higher sweat rate (r_s_ = 0.79, *p* = 0.006, Supplementary Figure S2B). A trend with a large effect size was found for sodium concentration by sex, with female athletes having lower concentrations compared to male athletes (494 (260–869) versus 1445 (667–1530) mg/h, *p* = 0.12, r = 0.54, Figure 1E). For all other comparisons, no differences were found.

## 4. Discussion

This is the first study to measure sweat and thermal response parameters in elite athletes with SCI across a variety of intensity levels, demographics, and sports characteristics. Differences in local sweat response [18] and exercise position compared to non-disabled athletes should be considered when designing a study protocol in athletes with SCI. Based on the findings of our study, the scapula or dorsal forearm appears to be the optimal location for sweat collection in wheelchair athletes. If access to a wheelchair-accessible scale is available, it is feasible to perform sweat and thermal response measurements on athletes with SCI in the field.

As the primary objective of this study was to investigate the feasibility of collecting thermoregulatory response data in the field, data were collected at a wide range of exercise intensities. Therefore, direct comparisons with other studies are difficult to make. However, the average and difference in Tc in our athletes are similar to those reported in other athletes with SCI [12,19,20,21,22]. Values for skin temperature were slightly lower, but also similar to those reported in other athletes with SCI [23,24]. A higher maximum Tc and a greater increase in Tc have been reported in individuals and athletes with tetraplegia and high-level paraplegia (lesion level T1–T6) compared to those with low-level paraplegia (lesion level below T6) or non-disabled athletes [4,20,21,25]. Similarly, the increase in skin temperature in low-level paraplegia corresponds to that of non-disabled individuals, whereas differences in progression have been found in individuals with tetraplegia and high-level paraplegia [23,25,26]. Perhaps because of our small sample size, which consisted primarily of male athletes with complete paraplegia measured during performance tests, we found no difference in Tc or skin temperature for the different subgroups. Furthermore, the intensity of the exercise may not have been high enough to show differences between subgroups, as another study that looked at the increase in Tc after 30 min of moderate-intensity exercise also found no increase in Tc in individuals with tetraplegia [11].

We found a maximum relative BM difference of 1.6%, which is still well below the 3% above which performance can be impaired [27,28]. Higher fluid losses can be expected during longer sessions, especially in hot conditions. Besides affecting performance, dehydration may also increase the risk of urinary tract infections, for which athletes with SCI are already at increased risk [29,30]. Secondary complications of urinary tract infections include fever and general malaise, and may affect performance [30].

Most team/skill athletes were dehydrated before the measurements but seemed to improve their hydration status during exercise. The reverse was true for the endurance athletes, most of whom were hydrated at the start but apparently did not hydrate well during exercise and hence were dehydrated afterward. For lesion characteristics, no differences in hydration status were observed. Our endurance athletes had a higher sweat rate compared to the team/skill athletes. Controversially, fluid intake tended to be lower in our endurance athletes. In particular, these athletes benefit from optimizing their fluid intake as they often need to perform for longer periods of time [31]. Corroborating previous findings [4], the sweat rate was low and even negative in our athletes with tetraplegia and in team/skill athletes. The reason for this may be twofold [25,32]. On the one hand, individuals with tetraplegia or complete lesions have little to no sweat production below but also above the lesion level, leading to lower sweat rates per se [33]. On the other hand, these athletes may experience thermal discomfort during exercise, such as feeling hot, which may lead them to drink more or even too much. This could lead to overhydration and low blood sodium concentrations. We observed this phenomenon particularly in team/skill athletes, where regular rest periods provide an opportunity to drink. Looking at the two studies in wheelchair basketball and rugby players measured during a game, fluid intake was comparable to our team/skill athletes measured during practice, but the sweat rate was much lower [12,19]. This underscores the need to sensitize and educate athletes with SCI about their hydration needs, both in terms of quantity and content. Adding electrolytes to their drinks may be an option as well.

We found a positive correlation between sweat rate and local electrolyte concentration in athletes with paraplegia, which is consistent with findings in non-disabled individuals [34]. Besides individual sweat rate, many other factors can influence electrolyte concentrations including exercise and environmental characteristics, as well as individual characteristics [16]. We found a trend toward lower electrolyte concentrations in female athletes, whereas electrolyte concentrations in recreational non-disabled athletes were similar between the sexes [10]. Consistent with previous findings in non-disabled athletes, we found that electrolyte concentrations are dependent on anatomical location [16]. Therefore, it is important to collect data from the same location when comparing multiple measurements from the same athlete.

The results of this study highlight the complexity of the sweat response in athletes with SCI. As sweat rate and electrolyte concentrations already show large variability among non-disabled athletes [16], personalized hydration strategies are even more relevant for athletes with SCI due to the added complexity of lesion characteristics [4,6]. In addition to educating athletes and their coaches about hydration, monitoring hydration status during regular training sessions could be implemented, as USG can be easily measured in the field. Intervention studies are urgently needed to investigate the effects of hydration status and sweat rate under various environmental conditions and exercise intensities, on the performance of athletes with SCI.

## 5. Strengths and Limitations

This study showed that it is feasible to perform field measurements of sweat response in elite athletes with SCI. Access to a wheelchair-accessible scale is crucial for accurate measurements. Data were collected from a diverse group of athletes during training and exercise testing.

To explore the possibility of field testing, we selected the most practical measurement solutions. Electrolyte concentration was measured using patches rather than the more accurate whole-body washdown method [16]. Sweat rate was estimated from the BM difference and fluid consumption. For a more accurate calculation, changes in BM due to metabolic mass loss and respiratory water loss should also be distinguished [16,35]. Furthermore, BM was measured while wearing (minimal) clothing, which may have underestimated the sweat rate we found [16,35]. Nevertheless, since more accurate measurements, including nude BM measurement, are not practical in the field, we tried to minimize these limitations by having the athletes wear only minimal clothing and towel-dry them before weighing.

To improve performance, athletes with SCI—especially those with a lesion above T6—may induce autonomic dysreflexia, a phenomenon also known as boosting. Because boosting is dangerous and potentially life-threatening, it is banned by the International Paralympic Committee [36]. Autonomic dysreflexia may also affect the response to thermal stress. Blood pressure is often used as an indicator of autonomic dysreflexia [37] but was not assessed in our study. Nevertheless, athletes were asked to void their bladder before the start of the exercise. Furthermore, our study team, which works with these athletes regularly, did not observe any significant heart rate deviations or other observations that would indicate autonomic dysreflexia in our athletes during the measurements. This supports that the responses measured in our study were not due to autonomic dysreflexia but rather to thermal stress.

This study was designed as an exploratory study, to investigate the feasibility of objective measurements in the field. As the pool of athletes with SCI competing at a(n) (inter)national level in Switzerland is limited, only a small number of athletes could be included in this study. Furthermore, the study population showed a large variation in individual characteristics and exercise intensities. Nevertheless, we believe that the results provide an interesting insight into the sweat response of athletes with SCI and that the differences investigated have clinical significance.

## Figures and Tables

**Table 1:** Athlete characteristics.

	Overall (n = 15)	Females (n = 3)	Males (n = 12)
**Age (years)**	30 (28–36)	36 (25–37)	30 (28–35)
**Height (cm)**	174 (160–183)	170 (150–171)	175 (163–185)
**Body mass (kg)**	66 (56–74)	48 (47–49)	69 (62–76)
**BMI (kg/m^2^)**	22 (20–24)	17 (16–21)	23 (21–24)
**Lesion cause (n (%))**			
** Traumatic SCI**	10 (67)	2 (67)	8 (67)
** Spina bifida**	5 (33)	1 (33)	4 (33)
**Lesion duration (years)**	22 (6–28)	23 (8–25)	16 (6–30)
**Lesion level (n (%))**			
** Tetraplegia**	3 (20)	0	3 (25)
** Paraplegia**	12 (80)	3 (100)	9 (75)
**Motor impairment (n (%))**			
** Complete**	11 (73)	2 (67)	9 (75)
** Incomplete**	4 (27)	1 (33)	3 (25)
**Time active at elite athlete level (years)**	3 (1–8)	3 (1–6)	4 (1–9)
**Sport type (n (%))**			
** Cycling**	9 (60)	2 (67)	7 (58)
** Wheelchair racing**	1 (7)	1 (33)	0
** Basketball**	2 (13)	0	2 (17)
** Rugby**	2 (13)	0	2 (17)
** Alpine skiing**	1 (7)	0	1 (7)
**Average weekly training**			
** Duration (hours)**	11 (9–12)	11 (10–20)	11 (9–12)
** Frequency (sessions)**	6 (5–7)	7 (6–12)	6 (5–7)

BMI = body mass index, SCI = spinal cord injury. Data are presented as median (Q1–Q3) unless indicated otherwise.

**Table 2:** Exercise, thermal, and sweat response parameters. Data are presented as median (Q1–Q3).

(**A**)				
		**Overall**	**Sport Type**	
			**Endurance (n = 10)**	**Team/Skill (n = 5)**
**Core temperature (°C)**				
** Average**		37.7 (37.3–37.8)	37.7 (37.6–37.8)	37.3 (37.0–38.1)
** Minimum**		37.1 (36.9–37.1)	37.1 (37.0–37.1)	36.6 (36.1–37.2)
** Maximum**		38.1 (37.9–38.6)	38.2 (38.1–38.6)	37.8 (37.4–38.9)
**Heart rate (bpm)**				
** Average**		126 (100–146)	134 (125–148)	100 (96–104)
Relative to max heart rate (%) *		73 (69–76)	74 (71–76)	71 (62–74)
** Minimum**		68 (61–74)	69 (63–83)	62 (61–72)
** Maximum**		178 (133–195)	190 (178–195)	133 (119–149)
**Urine specific gravity (g/mL)**				
** Pre-exercise**		1.020 (1.015–1.027)	1.017 (1.015–1.022)	1.028 (1.024–1.030)
** Post-exercise**		1.018 (1.014–1.026)	1.018 (1.014–1.026)	1.019 (1.016–1.029)
**Body mass difference**				
** Absolute (kg)**		0.20 (0.10–0.30)	0.30 (0.20–0.40)	−0.45 (−0.55–0.15)
** Relative (%)**		0.37 (0.12–0.61)	0.48 (0.34–0.63)	−0.63 (−0.82–0.19)
**Fluid intake (L/h)**		0.38 (0.28–0.54)	0.31 (0.21–0.44)	0.54 (0.43–0.61)
**Sweat rate (L/h)**		0.44 (0.35–0.63)	0.52 (0.42–0.73)	0.12 (−0.13–0.33)
(**B**)				
	**Sodium** **Concentration (mg/L)**	**Sodium Loss** **(mg/h)**	**Potassium** **Concentration** **(mg/L)**	**Potassium Loss** **(mg/h)**
**Head (n = 9)**	2100 (1300–2400)	1256 (584–1752)	210 (200–265)	121 (104–145)
**Scapula (n = 11)**	1700 (1400–2000)	876 (494–1460)	200 (160–220)	98 (74–131)
**Forearm (n = 10)**	1350 (1200–1800)	817 (424–1241)	240 (190–290)	107 (102–117)
**Shin (n = 5)**	1400 (770–1800)	920 (401–1022)	260 (180–300)	129 (101–153)

* max heart rate as defined during a previous performance test. There were no differences in electrolyte concentrations measured on the left and right sides of the body (*p* ≥ 0.12, r ≤ 0.70). Therefore, only data from the side with the most available data points are presented: scapula (left), arm (left), or shin (right).

## Data Availability

The dataset generated and analyzed in the current study is not publicly available but is available from the corresponding author upon reasonable request.

## Supplementary Materials

The following supporting information can be downloaded at https://www.mdpi.com/article/10.3390/sports12030081/s1, Table S1: STROBE checklist of items that should be included in reports of cross-sectional studies; Table S2: Core and skin temperature as measured using a wearable sensor; Figure S1: Overview of the location of the absorbent patches for sweat collection (A), and sensors for heart rate (B) and core temperature measurements (C); Figure S2: Scatter plots for sweat rate versus A) average heart rate (r_s_ = 0.61, p = 0.021) and B) sodium concentration at the left scapula versus (r_s_ = 0.79, p = 0.006).
